# A191 PERSISTENT BENEFIT OF DIETITIAN-LED GLUTEN-FREE DIET EDUCATION AT CD DIAGNOSIS ON DIETARY ADHERENCE IN CHILDREN AND ADULTS WITH TYPE 1 DIABETES AND CELIAC DISEASE

**DOI:** 10.1093/jcag/gwab049.190

**Published:** 2022-02-21

**Authors:** P Marcon, A Clarke, K Pace, C McDonald, F Saibil, H A Lochnan, Z Punthakee, F Mahmud

**Affiliations:** 1 GI, The Hospital for Sick Children, Toronto, ON, Canada; 2 Endocrinology, The Hospital for Sick Children, Toronto, ON, Canada; 3 Western University Faculty of Science, London, ON, Canada; 4 Sunnybrook Health Sciences Centre, Toronto, ON, Canada; 5 Ottawa Hospital, Ottawa, ON, Canada; 6 McMaster University Faculty of Health Sciences, Hamilton, ON, Canada

## Abstract

**Background:**

Celiac disease (CD) is a common autoimmune comorbidity of type 1 diabetes (T1D) with a gluten-free diet (GFD) being the current gold standard treatment for this condition. Adherence to a GFD can be impacted by several factors including dietetic counselling, yet little is known about the impact of clinic-based interventions on long-term GFD adherence in this population.

**Aims:**

To prospectively evaluate the impact of a dietitian-led GFD education intervention on adherence to a GFD in children and adults with T1D and CD over a 3-year period.

**Methods:**

A cohort of N=62 pediatric and adult subjects who screened seropositive for CD as part of the CD-DIET clinical trial were followed over a 3-year period post-CD diagnosis and assessed on the basis of the GFD education regimen they received at initial CD diagnosis. This included 3 groups: 1) intensive dietitian training (IDT = 5 dietitian visits over 1 year while following GFD), 2) single dietitian training (SDT = 1 GFD training session after 1 year of following GCD) and 3) no dietitian training (NDT) at CD diagnosis. Annual visits included serologic testing of TTG-IgA titres, anthropometric assessments and the completion of questionnaires evaluating diet and adherence to a GFD. Data was analysed longitudinally using linear mixed effects and generalized estimating equations (GEE) regression modeling adjusting for the fixed effects of age, sex, duration of diabetes and time.

**Results:**

At baseline, participants who received IDT (n=15), SDT (n=16) and NDT (n=31) represented 24.2%, 25.8%, and 50.0% of the cohort, respectively. Over the 3-year study period, participants in the IDT group had the greatest odds of self-reporting being a GFD, with odds 4.3 (95%CI: 1.1 to 16.4; P=0.033) and 9.5 (95%CI: 2.7 to 33.7; P<0.001) greater than the SDT and NDT groups, respectively. The assessment of daily gluten intakes less than 10mg, as recommended for a GFD, revealed a lack of differences between the IDT and SDT groups. In contrast, the NDT group had significantly lower odds of meeting this threshold relative to those who received IDT (OR=0.2; 95%CI: 0.04 to 0.56; P=0.004). No longitudinal differences in TTG-IgA levels were seen between groups over the 3-year period.

**Conclusions:**

In diabetes patients greater contact with a dietitian at CD diagnosis was associated with higher levels of GFD adherence over time, which was not reflected in follow-up Serologic evaluation. These findings highlight the importance of nutritional support in patients with both diabetes and celiac disease at the time of CD diagnosis. In addition, following TTG-IgA alone does not fully inform dietary compliance to a GFD.

Diet teaching stratification

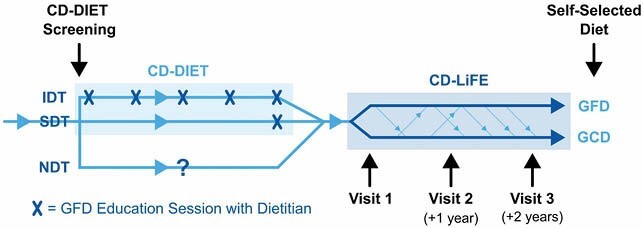

Diet assignment

**Funding Agencies:**

Juvenile Diabetes Research Foundation / PSI

